# “中国乌蒙山肺癌”：云贵高原燃煤污染相关区域性肺癌的证据链、临床及分子特征

**DOI:** 10.3779/j.issn.1009-3419.2026.102.16

**Published:** 2026-06-20

**Authors:** 中国西部肺癌研究协作中心

**Keywords:** 中国乌蒙山区, 区域性肺癌, 宣威, 富源, 家庭燃煤, 室内空气污染, 多环芳烃, 家族肺癌, Wumeng Mountain Area of China, Regional lung cancer, Xuanwei, Fuyuan, Household coal combustion, Indoor air pollution, Polycyclic aromatic hydrocarbons, Familial lung cancer

## Abstract

**背景与目的:**

长期家庭燃煤、煤源和矿物组分差异、能源结构及社会经济条件共同构成肺癌区域暴露基础。本研究对乌蒙山地区燃煤污染与“中国乌蒙山肺癌”的相关性进行多断面、多维度和多组学研究，并探讨“中国乌蒙山肺癌”概念边界、证据链、致癌组分、临床和分子特征以及“中国乌蒙山肺癌”精准防控路径，以期为“中国乌蒙山肺癌”高发地区肺癌一级、二级、三级和四级预防，以及高危人群筛查和分子分型管理提供证据和参考。

**方法:**

在我们前期研究建立的较完整的证据链，涵盖家庭煤烟暴露、煤源差异、多环芳烃（polycyclic aromatic hydrocarbons, PAHs）、炉灶干预、非吸烟女性肺癌和肿瘤多组学特征的基础上，应用叙述性综述方法进行文献检索和证据整合。检索数据库包括PubMed、Web of Science、Embase、中国知网（CNKI）和万方数据库，时间范围为各数据库建库至2026年5月；检索词包括：Xuanwei、Fuyuan、Wumeng Mountains、household coal combustion、smoky coal、lung cancer、PAHs、methylated PAHs、stove improvement、never-smoking women、germline susceptibility、microbiome和multi-omics。证据整合采用“暴露-污染组分-生物效应-临床分子特征-防控转化”证据链。

**结果:**

（1）PAHs/甲基化PAHs、细颗粒物2.5（particulate matter 2.5, PM_2.5_）/黑碳、无机矿物/金属、羰基化合物与混合暴露为燃煤污染相关肺癌主要致癌组分；（2）高发于非吸烟女性、腺癌占比高、低发病年龄，易早期转移、多发肺癌病灶以及家族发病聚集是乌蒙山地区燃煤污染相关肺癌的临床特征；（3）表皮生长因子受体（epidermal growth factor receptor, *EGFR*）总突变较高，但经典*EGFR* 19外显子缺失和L858R突变比例相对较低，而*EGFR* G719X、S768I突变、Kirsten大鼠肉瘤病毒癌基因同源物（Kirsten rat sarcoma viral oncogene homolog, *KRAS*）G12C突变，以及间变性淋巴瘤激酶（anaplastic lymphoma kinase, *ALK*）和ROS肉瘤致癌因子1-受体酪氨酸激酶（c-ROS proto-oncogene 1, receptor tyrosine kinase, *ROS1*）基因融合突变显著高于非中国乌蒙山肺癌；（4）胚系突变基因*ARHGEF5*、*MYO18B*和*MUC16*为乌蒙山地区燃煤污染相关肺癌的潜在遗传易感基因，它们与端粒长度维护基因*POT1*、*TERT*和*TERF2*交互作用，并可能与该地区肺癌家族聚集存在密切关系；（5）室内燃煤污染导致痰液微生物群改变，以及呼吸道长期慢性感染共同影响呼吸道免疫微环境和污染物代谢相关通路异常，增加肺癌风险。

**结论:**

“中国乌蒙山肺癌”是与室内外燃煤污染相关肺癌，具有好发于非吸烟女性、高腺癌比、低发病年龄、肺部多病灶，特殊的呼吸道慢性感染、污染物代谢和免疫微环境，以及特殊的驱动基因和胚系突变基因突变谱等临床和多组学特征。

## Abstract (en)

**Background and objective:**

Long-term household coal burning, differences in coal sources and mineral compositions, energy structure, and socioeconomic conditions jointly constitute the regional exposure basis for lung cancer. This study conducts a multi-sectional, multi-dimensional and multi-omics investigation on the correlation between coal-burning pollution in the "Wumeng Mountain region" and "China Wumeng Mountain lung cancer", and explores the concept boundaries, evidence chain, carcinogenic components, clinical and molecular characteristics, as well as the precise prevention and control pathways of "China Wumeng Mountain lung cancer", with the aim of providing evidences and references for the primary, secondary, tertiary and quaternary prevention of lung cancer in high-incidence areas of "China Wumeng Mountain lung cancer", as well as the screening and molecular typing management of high-risk populations.

**Methods:**

Based on the relatively complete evidence chain established in our previous research, covering household coal smoke exposure, coal source differences, polycyclic aromatic hydrocarbons (PAHs), stove intervention, lung cancer and tumor multi-omics characteristics, a narrative review method was applied for literature search and evidence integration. The search databases included PubMed, Web of Science, Embase, China National Knowledge Infrastructure (CNKI) and Wanfang Database, with the time range from the establishment of each database to May 2026; search terms included: Xuanwei, Fuyuan, Wumeng Mountains, household coal combustion, smoky coal, lung cancer, PAHs, methylated PAHs, stove improvement, never-smoking women, germline susceptibility, microbiome and multi-omics. The evidence integration adopted the "exposure-pollutant component-biological effect-clinical molecular characteristics-prevention transformation" evidence chain.

**Results:**

(1) PAHs/methylated PAHs, particulate matter 2.5 (PM_2.5_)/black carbon, inorganic minerals/metals, carbonyl compounds and mixed exposure are the main carcinogenic components of coal-burning pollution-related lung cancer; (2) It is prevalent among non-smoking women, with a high proportion of adenocarcinoma, low age of onset, easy early metastasis, multiple lung lesions and family aggregation are the clinical characteristics of coal-burning pollution-related lung cancer in the Wumeng Mountain region; (3) The total epidermal growth factor receptor (*EGFR*) mutation is relatively high, but the proportions of classic *EGFR* 19 exon deletion and L858R mutation are relatively low, while *EGFR*-G719X, S768I mutations, Kirsten rat sarcoma viral oncogene homolog (*KRAS*) G12C mutations, and anaplastic lymphoma kinase (*ALK*) and c-ROS proto-oncogene 1, receptor tyrosine kinase (*ROS1*) gene fusion mutations are significantly higher than those in non-Wumeng Mountain lung cancer; (4) Germline mutation genes *ARHGEF5*, *MYO18B* and *MUC16* are potential genetic susceptibility genes for coal-burning pollution-related lung cancer in the Wumeng Mountain region, and they interact with telomere length maintenance genes *POT1*, *TERT* and *TERF2*, and may have a close relationship with the existence of lung cancer family aggregation in this region; (5) Indoor coal-burning pollution leads to changes in the microbial community of sputum, and long-term chronic infection of the respiratory tract jointly affect abnormal pathways related to the immune microenvironment and pollutant metabolism, increasing the risk of lung cancer.

**Conclusion:**

China Wumeng Mountain lung cancer is a type of lung cancer related to indoor and outdoor coal-burning pollution, with a predilection for never-smoking women, high proportion of adenocarcinoma, low age of onset, multiple lung lesions, as well as special chronic respiratory infections, abnormal pollutant metabolism and immune microenvironment, as well as special driver gene and germline mutation gene mutation spectra.

## 1 家庭燃煤污染与肺癌相关性的全球证据

肺癌病因研究长期以烟草暴露为核心，但在不吸烟或低吸烟率人群中，家庭空气污染、职业和环境暴露、慢性肺部疾病，以及遗传易感性同样重要。家庭燃烧煤炭和其他固体燃料可产生颗粒物、多环芳烃（polycyclic aromatic hydrocarbons, PAHs）、挥发性有机物、金属和无机矿物质颗粒等复杂污染物。国际癌症研究机构（International Agency for Research on Cancer, IARC）已将家庭煤炭燃烧产生的室内排放物列为对人类致癌的1类致癌物^[1]^。

除IARC致癌性评价外，系统综述和*meta*分析^[2]^显示，家庭燃煤与肺癌风险升高密切相关，且效应强度存在明显地区差异。另一项系统综述和*meta*分析^[3]^表明，使用煤炭、木材、木炭、农业废弃物、动物粪便或煤油等污染性燃料造成的家庭空气污染，与慢性阻塞性肺疾病（chronic obstructive pulmonary disease, COPD）、肺癌、脑血管疾病和缺血性心脏病风险升高密切相关。

中国云贵高原乌蒙山区横跨云南、贵州和四川，毗邻山区，当地煤炭资源丰富，地质来源独特且分布广泛。居民长期用本地煤炭作为取暖和烹饪燃料，形成持续的家庭燃煤空气污染暴露。以宣威-富源为核心证据区，我们前期研究已建立较完整的燃煤污染相关肺癌证据链，涵盖家庭煤烟暴露、煤源差异、PAHs、炉灶干预、非吸烟女性肺癌和肿瘤多组学特征。云南昭通、曲靖、昆明及贵州毕节、六盘水、黔西南布依族苗族自治州部分地区与宣威-富源位于乌蒙山区，在地理环境、能源结构和生活燃煤背景方面具有高度相似性，可作为区域扩展参照，但仍需统一暴露评价和癌症登记资料验证。

乌蒙山区是云贵高原重要的高原山区和煤炭资源分布区域。广义乌蒙山区及其毗邻地区人口较多，乡村人口比例较高。宣威、富源和盘州（原盘县）等地在肺癌研究中尤为重要，具体县域划分及证据边界见表1。我们早期研究主要聚焦宣威，随后宣威-富源燃煤暴露、非吸烟女性肺癌和煤源差异研究不断深入^[4-14]^。随着肺癌筛查、临床病例登记和流行病学调查的推进，周边部分地区也逐渐成为值得进行肺癌高发人群进一步验证的扩展参照区域^[15-18]^。

**表1:** 中国乌蒙山区燃煤污染相关肺癌的证据链与证据等级

Evidence link	Representative evidence	Core region/ study population	Evidence category	Current limitations
Elevated epidemiological incidence	Long-term mortality trends and spatial heterogeneity in Xuanwei; county-level incidence registration and village-level mortality spatial clustering in Fuyuan	Xuanwei-Fuyuan	Strong	Insufficient data from extension areas; core area requires continued registration improvement
Stove intervention	Improved stoves/ventilation reduce exposure and risk	Xuanwei	Strong	Insufficient reassessment following clean-energy transition in recent years
Coal-source heterogeneity	Differential risk by smoky-coal subtype and geological coal deposit	Xuanwei-Fuyuan	Strong	Insufficient coal-source comparison data from extension reference areas
PAHs/ methylated PAHs	5-MC; pre-age-18 exposure	Xuanwei	Strong	Multi-pollutant collinearity remains to be addressed
Inorganic minerals/metals	Quartz, nano-scale iron-bearing particles, iron-rich particles, iron-bearing chlorite-group minerals, and resuspended coal-ash nano-minerals	Xuanwei-Fuyuan	Moderate	Independent carcinogenic contribution and exposure pathways require quantification
Tumor omics	Mutational signatures, proteogenomic subtypes	Xuanwei	Strong/moderate	Sample size and out-of-region validation insufficient
Host susceptibility	Familial clustering, germline mutations, pulmonary microbiome-host expression, and host-defense clues	Wumeng Mountain familial lung cancer cases and adjacent normal lung tissues	Moderate/exploratory	Causal mechanisms require further validation

PAHs: polycyclic aromatic hydrocarbons; 5-MC: 5-methylchrysene.

本文在整合宣威-富源核心证据区及扩展参照区研究的基础上，提出“中国乌蒙山肺癌”这一概念及其研究视角，并梳理云贵高原燃煤污染相关区域性肺癌的证据链、边界和研究进展。在这一视角下，长期家庭燃煤、煤源和矿物组分差异、能源结构及社会经济条件共同构成区域暴露基础。本研究将“中国乌蒙山肺癌”作为“乌蒙山区燃煤污染相关区域性肺癌”的概念性简称，强调其并非新的病理类型或正式疾病名称，而是整合区域暴露、流行病学、临床表型、宿主易感性和多组学证据的研究框架。

## 2 文献检索、证据评价与证据整合

### 2.1 检索方法

本文采用叙述性综述方法进行文献检索和证据整合。检索数据库包括PubMed、Web of Science、Embase、中国知网（CNKI）和万方数据库，时间范围为各数据库建库至2026年5月。英文检索词包括Xuanwei、Fuyuan、Wumeng Mountains、household coal combustion、smoky coal、lung cancer、PAHs、methylated PAHs、stove improvement、never-smoking women、germline susceptibility、microbiome和multi-omics；中文检索词包括宣威、富源、乌蒙山、家庭燃煤、烟煤、肺癌、多环芳烃、炉灶改良、非吸烟女性、家族肺癌、遗传易感、微生物组和多组学等。

### 2.2 纳入文献

主要包括与宣威、富源及云贵高原相关产煤、用煤地区有关的流行病学研究、暴露评价、环境毒理、炉灶干预、临床病理、驱动基因、表观遗传、微生态、肿瘤多组学和区域防控研究。排除仅为个案报道、缺乏区域暴露信息、无法追溯原始数据来源或与家庭燃煤暴露无直接关系的文献；对综述、网页或报告类资料，仅用于背景说明，不作为证据等级判定的主要依据。

### 2.3 证据整合

采用“暴露-污染组分-生物效应-临床分子特征-防控转化”的证据链思路。本文未进行定量*meta*分析，证据等级用于叙述性评价而非GRADE系统判定。Strong（强）定义为：具有重复人群研究或长期队列/病例对照证据，暴露评价较清楚，并有干预、剂量反应、组分检测或组学证据支持；Moderate（中等）定义为：有多项观察性或实验室证据支持，但样本量、区域覆盖、暴露量化或外部验证不足；Exploratory（探索性）定义为：主要来自单项研究、医院病例、家族亚群、有限组学样本或机制假说，尚需独立验证。

基于上述原则，表1中的证据等级主要反映证据链完整度、区域可重复性和因果解释强度。宣威-富源相关长期流行病学、煤源差异、PAHs/甲基化PAHs、炉灶干预和部分肿瘤组学证据被归为较强证据；无机矿物/金属、共病现象、宿主易感性、微生态和黏蛋白16（mucin 16, MUC16）/纤毛功能相关内容更多被视为中等或探索性线索。

## 3 结果

### 3.1 “中国乌蒙山肺癌”的概念：从“宣威肺癌”到云贵高原区域性肺癌

本研究所称“中国乌蒙山肺癌”，是“乌蒙山区燃煤污染相关区域性肺癌”的概念性简称，指发生于云贵高原乌蒙山区及其毗邻产煤、用煤地区，与长期家庭燃煤导致环境空气严重污染密切相关肺癌高发，表现出区域聚集、特定暴露背景和一定临床分子特征的肺癌病例群体。它不是临床诊断名称，也不等同于新的病理类型；其价值在于为区域暴露、流行病学、临床表型、宿主易感性和分子机制研究提供统一的肺癌高发人群研究现场。

既往文献中“宣威肺癌”的使用并不完全一致，有时指宣威本地肺癌，有时实际上涵盖富源、盘州等相邻燃煤高发地区。为避免概念外推，本研究采用表2^[19-21]^所示的三层区域边界，并根据证据链完整程度区分核心证据区、扩展参照区和待验证区。暴露评价以家庭燃煤空气污染为核心，临床表型主要关注非吸烟女性、早发、腺癌比例较高、多发肺结节和家族聚集等特征，分子证据则包括驱动基因谱、突变特征，表观遗传改变和宿主防御相关信息。共病现象可作为探索性研究方向，但不作为该概念的定义性特征。

**表2:** 中国乌蒙山区燃煤污染相关肺癌的三层区域边界

Tier	Representative regions	Evidence strength	Inclusion criteria	Usage boundary
Core evidence area	Xuanwei-Fuyuan	Strong	Relatively complete long-term epidemiological data, coal-source heterogeneity, PAHs/methylated PAHs, stove-intervention studies, and multi-omics evidence	Primary evidence base for conceptual formulation and mechanistic inference
Extension reference area	Yunnan: Zhaotong (Zhenxiong, Zhaoyang, Ludian, Daguan, Yiliang, Weixin), Qujing (Qilin, Zhanyi, Malong, Huize), Kunming (Xundian, Dongchuan); Guizhou: Bijie (Weining, Hezhang, Nayong), Liupanshui (Panzhou, Shuicheng, Liuzhi); Qianxinan (Xingyi)	Moderate/requires strengthening	Substantial geographic, energy-structure, and domestic coal-use similarities with Xuanwei-Fuyuan; some areas have screening, survey, or clinical case data	Regional extension reference; inferences not at same strength as core area
Area pending validation	Other counties across the broader Wumeng Mountain region with similar coal-exposure backgrounds	Weak/pending validation	Potential coal production, use, and household exposure, but lacking systematic cancer registry, exposure assessment, and omics evidence	Future research targets; not classified as confirmed high-incidence areas

Fuyuan administratively belongs to Qujing city. However, due to the relatively complete existing evidence, this study includes it in the core evidence area. When listing the expanded reference areas for Qujing, they are not included repeatedly. The direct evidence in Fuyuan includes the county-level disease registration^[19]^ (for details, see Part Four), the spatial aggregation of lung cancer deaths at the administrative village level, the association of coal source distribution^[20]^, and the clinical follow-up data of non-smoking women from Xuanwei to Fuyuan area^[21]^.

### 3.2 乌蒙山区域暴露背景：高原山区、煤源差异、家庭燃煤与室内外暴露

对乌蒙山区肺癌的研究需要同时考虑地理、气候、能源、产业和家庭生活方式。高原山区地形、分散的村寨或自然村民居住模式以及寒潮和低温天气，均可能增加居民烹饪、烧水和取暖等生活用煤需求。同时，煤炭开采、运输、储存和农村家庭长期用煤，使居民既暴露于厨房和居室内煤烟，也可能持续接触村庄户外空气中的燃煤污染物。与单纯城市空气污染不同，乌蒙山区燃煤空气污染是高原寒冷环境、煤炭资源分布、农业人口，经济相对滞后和家庭长期用煤共同作用的结果。

燃煤空气污染不仅发生在厨房和居室内。需要区分的是，家庭生活燃煤与工业燃煤排放并不完全相同。李继华等^[22]^比较滇东工业燃煤区与宣威室内生活燃煤区后指出，两个地区的肺癌流行病学特征存在显著差异；除家庭燃煤外，工业生产排放也可能构成周边居民不可忽视的环境暴露。煤矿职业暴露同样需要纳入评价。Hosgood等^[23]^在宣威男性人群病例对照研究中发现，在调整吸烟和家庭用煤等因素后，煤矿工人肺癌风险仍然升高；工作10年以上者肺癌风险更为突出。

农村家庭煤炉、火塘和取暖设施可造成室内高浓度污染；煤炭开采、运输、储存，以及邻里燃煤排放，还可能使煤烟扩散到村庄户外空气中，使居民在离开厨房和居室后仍持续接触燃煤污染物^[24-27]^。滇东农村环境流行病学调查^[16,18]^显示，高发村居民使用烟煤和焦煤的比例较高，而对照村更多使用无烟煤和柴草，提示燃料类型是区域肺癌风险差异的重要证据。区域评价应同时研究家庭炉具、煤源、职业、工业排放和居住史。

高海拔寒冷环境可能主要通过延长取暖季、增加室内停留时间和生活用煤需求，间接放大家庭燃煤污染暴露。至于低氧微环境是否直接影响肺癌易感性，仍需进一步研究。因此，本研究将高海拔寒冷环境理解为可加重燃煤暴露的背景条件，而非与燃煤污染并列的已证实病因。

在长期燃煤暴露和相对稳定的聚居环境下，家族聚集、非吸烟女性肺癌、多发病灶和区域相关分子特征，可能共同反映环境暴露与宿主易感性之间的复杂关系（图1）。需要强调的是，暴露背景相似只能支持区域扩展假说，不能替代人群发病资料、个体暴露评价和分子组学证据。因此，宣威-富源仍是核心证据区，周边相似煤区仍属于扩展参照区或待验证区。

**图1:**
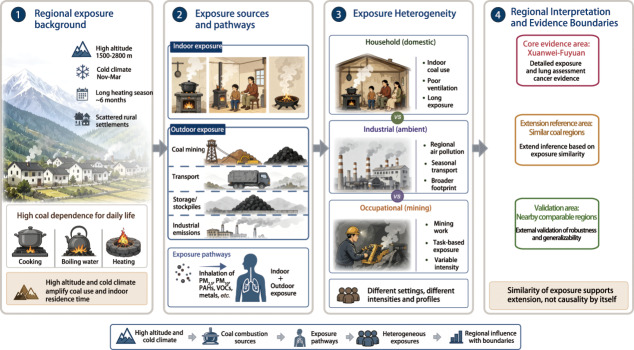
中国乌蒙山区燃煤污染相关肺癌的区域暴露背景与暴露路径。高原寒冷气候、分散村寨居住模式及较高的生活用煤需求共同构成该区域的暴露背景；家庭室内燃煤、煤炭开采、运输与储存，以及工业排放，可形成室内外暴露路径。不同暴露场景下，污染强度及污染谱存在差异，区域解释应以既有证据边界为前提。

### 3.3 核心流行病学证据：宣威-富源队列、病例对照、炉灶干预与长期累积暴露

#### 3.3.1 宣威长期肺癌高发与空间区域异质性

宣威成为燃煤污染相关肺癌研究的经典自然人群研究现场，首先在于其长期家庭烟煤暴露背景清晰。多项研究^[4-11,28]^较早开展多学科调查，随后研究逐步将肺癌高发与室内燃煤排放、炉灶干预、煤源差异和个体PAHs暴露联系起来。

多项死亡登记、疾病负担和空间分析研究^[29-35]^显示，宣威肺癌死亡率长期处于高水平，且存在明显乡镇和村级空间异质性。2011-2013年男性和女性肺癌年龄标化死亡率分别为82.53/10万和62.62/10万，约分别为同期全国农村地区肺癌发病率和死亡率的3和6倍^[30]^。2016年疾病负担研究也显示，肺癌仍对当地居民健康造成显著影响。近年来部分指标虽有所下降，但总体负担仍较高（代表性人群证据见表3^[18-20,30,31,33]^）。

**表3:** 中国宣威-富源肺癌高发现场核心证据区主要人群证据及使用边界

Region	Period	Indicator	Key findings	Interpretive boundary	Ref.
Xuanwei	1990-2016	Long-term mortality registration	Crude mortality rose from 34.0 per 100,000 to 102.3 per 100,000; 87.2 per 100,000 in 2014-2016; Age-standardized mortality (2011-2013): male 82.53 per 100,000, female 62.62 per 100,000	Long-term high burden; partial recent decline	^[30,33]^
Xuanwei	2011-2015	Spatial heterogeneity	Annual crude mortality 70.78 per 100,000-80.30 per 100,000	Pronounced township- and village-level variation	^[31]^
Eastern Yunnan coal area	Screening period	Screening positivity rate	Adjusted: 763.08 per 100,000; world-standardized: 426.28 per 100,000	Screening metric, not equivalent to incidence	^[18]^
Fuyuan	2002-2004	World-standardized incidence	76.45 per 100,000	Supports elevated incidence; requires continued registration updates	^[19]^
Fuyuan	2010-2019	Crude lung cancer mortality	84.1 per 100,000; 6828 recorded deaths	Village-level spatial clustering corresponds with smoky-coal-subtype deposit distribution	^[20]^

The coal-producing (burning) area in Eastern Yunnan is the regional scope covered by the screening study, not a single administrative region. This indicator is not equivalent to the incidence rate in a county.

#### 3.3.2 富源纳入核心证据区的依据

富源不应仅被视为宣威的地理邻近地区。现有证据包括县域肺癌发病登记、2010-2019年死亡登记、行政村级死亡空间聚集、烟煤亚型矿床分布关联、滇东筛查调查，以及宣威-富源非吸烟女性临床随访资料^[12,18-21,36]^。这些证据支持将富源与宣威共同列为肺癌高发现场核心证据区；但与宣威相比，富源在炉灶干预和肿瘤多组学方面的证据仍相对不足（主要指标及统计口径见表3^[18-20,30,31,33]^）。

#### 3.3.3 煤源差异、替代性解释与炉灶干预

煤种和地质煤源差异是解释区域肺癌发病率和死亡率风险异质性的关键。不同烟煤亚型、地质煤层来源和早期生活阶段用煤经历等均与肺癌风险差异相关^[10,14,37-41]^。吸烟仍是需要独立控制的肺癌发病重要危险因素；高强度烟煤暴露可影响吸烟相对效应的观察，但不能据此弱化控烟^[42]^。后续研究应该进一步将煤源差异关联至具体污染物组分^[43,44]^。

氡和水土中部分重金属曾被作为替代解释进行检验。现有监测尚不足以支持其为当地肺癌异常高发的主要原因，但颗粒物携带矿物和金属参与混合暴露的可能性仍需进行更深入的研究^[14,45-48]^。

炉灶改良、烟囱安装和排烟条件改善与肺癌及COPD风险下降相关。个人和室内监测^[9,24,25,27,49]^进一步显示，通风炉具可显著降低PM_2.5_暴露。这些结果说明，该区域性肺癌负担具有明确的一级预防路径。

#### 3.3.4 非吸烟女性研究价值与证据体系

宣威女性主动吸烟率较低，但肺癌负担却较高，使核心区尤其适于观察家庭燃煤空气污染与肺癌高发相关性效应^[12,15,18]^。美国国立癌症研究院Qing Lan团队和香港大学Tian Linwei团队从流行病学、精准暴露评价、环境毒理和分子组学等方向推进了证据链^[9,10,13,14,24,25,28,43,44,49-56]^。相关研究共同支持将评价重点从“是否用煤”推进至“煤源-污染谱-生物效应-肺癌高发”证据体系。

### 3.4 乌蒙山区燃煤空气暴露的致癌组分

乌蒙山区燃煤空气暴露研究的核心问题是煤炭燃烧排放物中哪些组分驱动肺癌发生。与城市交通污染不同，核心证据区农村居民可同时接触室内高浓度煤烟、户外燃煤排放和不同地质煤源产生的污染物。燃煤污染的致癌效应通常不是由单一化合物所致，而是由有机污染物、颗粒物、无机矿物和反应性气体共同构成的混合暴露所驱动。因此，区域风险差异可能不仅取决于是否使用煤炭，更取决于煤源、炉具、通风条件和混合污染谱的组合。

第一类关键组分是PAHs及其衍生物。暴露评价研究^[14,28,43,57-59]^显示，使用烟煤者的个人和室内PAHs暴露通常高于使用无烟煤者，不同地质煤源也可形成差异明显的污染谱。近年研究进一步将暴露评价从“是否使用烟煤”推进到儿童期、青少年期至成年期的长期累积暴露。Portengen等^[43]^发现，18岁前暴露影响更为突出，甲基化PAHs，特别是5-甲基屈（5-methylchrysene, 5-MC），可能是解释煤源风险差异的重要组分。

第二类组分是细颗粒物2.5（particulate matter 2.5, PM_2.5_）、黑碳和超细颗粒。颗粒物可携带PAHs、金属和矿物组分进入人体细支气管和肺泡。模拟燃烧和现场监测研究^[24-26,37,38,60-62]^显示，煤种、炉具通风、季节、燃烧时间和村镇环境均会影响PM_2.5_、黑碳、二氧化氮（NO_2_）、二氧化硫（SO_2_）和PAHs水平；家庭燃煤排放还影响户外空气质量。单颗粒分析^[37,63,64]^进一步提示，燃煤排放中矿物颗粒、飞灰、烟炱聚集体和富硅、富铁颗粒较常见。

第三类组分是无机矿物和金属。煤地质、煤灰、环境介质和肺组织研究^[37-40,46-48,50,55,65-77]^提示，除石英外，纳米尺度或亚微米尺度含铁矿物颗粒、富铁颗粒及含铁绿泥石类矿物也是值得重点关注的致癌物。这里所称“纳米尺度含铁颗粒”并非单指单质铁纳米颗粒，而是指可携带或释放铁的细小矿物颗粒。Dai等^[50]^的研究发现，宣威煤中的绿泥石类矿物包括含铁的鲕绿泥石（chamosite）。Lu等^[38]^的研究进一步显示，宣威燃煤排放中的含铁矿物颗粒占已测颗粒的10%以上，亚微米颗粒因溶解态铁比例较高而具有更强的氧化潜能。Han等^[71]^在C1煤及肺癌、癌旁肺组织中识别出针状富铁（berthierine-chamosite），并观察到铁释放和组织蓄积线索。上述研究结果支持将富铁矿物作为混合暴露中的重要致癌组分，但其独立致癌贡献仍需进行更多的量化研究。铁死亡相关研究可为铁相关暴露、氧化应激与肺上皮损伤之间的联系提供探索性线索，但目前不能将组织铁死亡改变直接归因于外源含铁颗粒暴露^[78]^。

第四类组分是羰基化合物和气态污染物。家庭燃煤可产生甲醛、乙醛、NO_2_和SO_2_等刺激性或潜在促炎气体。相关研究^[56,79,80]^显示，不同燃料和炉具对应的气体水平存在明显差异；不同来源煤烟颗粒还能引起炎症反应和气道屏障损伤。

总体而言，PAHs和甲基化PAHs具有较强的直接致突变潜能，PM_2.5_和黑碳可作为污染物载体和剂量指标，无机矿物质与金属可能介导氧化应激和炎症反应，羰基化合物及NO_2_/SO_2_等气态污染物则可能损伤气道屏障（图2）。本研究所称“肺部内暴露”，是指外部环境中的煤烟污染物进入、沉积或滞留于肺部组织后，实际作用于肺上皮细胞和局部微环境的污染物剂量。代表性检测证据及其使用边界见表4^[14,24,25,28,38,43,50,55-58,61-71,78-80]^。未来扩展区研究不能仅比较“是否用煤”，还应同时评价煤源、炉具、通风和多污染物混合谱。

**图2:**
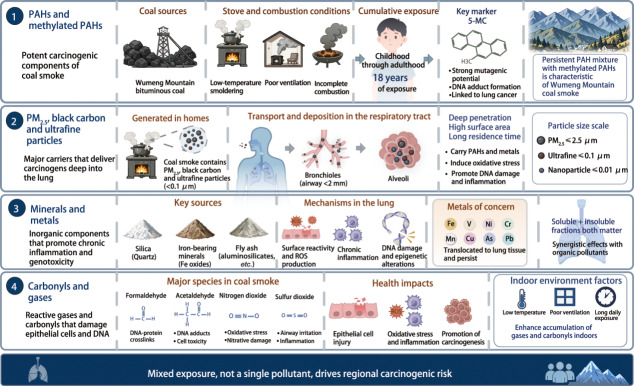
中国乌蒙山区燃煤污染相关肺癌的致癌组分与混合暴露特征。家庭燃煤、煤源差异及室内外暴露共同塑造该区域的混合暴露谱，主要包括PAHs/甲基化PAHs、PM_2.5_/黑碳、无机矿物/金属和羰基化合物等。与单一污染物相比，混合暴露更能解释区域肺癌致癌风险差异。

**表4:** 燃煤污染组分、代表性检测证据及研究意义

Component class	Representative evidence	Core findings	Usage boundary	Ref.
PAHs and methylated PAHs	Extended PAH analysis across coal sources, stove types, and exposure stages	Smoky coal and different geological sources produce distinct pollution profiles; pre-age-18 exposure and 5-MC warrant attention	Multi-pollutant collinearity must be addressed	^[14,28,43,57,58]^
PM_2.5_, black carbon, and ultrafine particles	Simulated combustion, personal and indoor/outdoor monitoring	Coal type, stove ventilation, season, and combustion time affect exposure; household emissions may affect outdoor air	Total PM_2.5_ does not capture the full pollution profile	^[24,25,61,62]^
Single-particle composition	SEM-EDX analysis of 796 particles; single-particle aerosol mass spectrometry identifying 9 particle classes	Mineral particles, fly ash, soot aggregates, and silicon- or iron-enriched particles are common; composition varies by particle size	Must be analyzed in conjunction with individual exposure and health outcomes	^[63,64]^
Quartz, nano-minerals, and coal ash	Coal geology, coal-ash resuspension, and personal air-sample studies	Coal-ash disturbance and resuspension may constitute supplementary exposure pathways	Quartz or nano-minerals cannot be designated as a single cause	^[50,55,65 -67]^
Iron-bearing minerals and tissue elements	Size-segregated particles, road dust, lung tissue elements, and oxidative-potential studies	Nano-scale iron-bearing, iron-rich, and iron-bearing chlorite-group minerals may contribute through iron release, oxidative stress, and inflammation	Independent carcinogenic contribution requires quantification	^[38,68-71]^
Ferroptosis-related clues	Ferroptosis-related protein studies in Xuanwei NSCLC tissue	May serve as exploratory mechanistic clues linking iron-associated particles to tumorigenesis	Cannot serve as environmental causal evidence	^[78]^
Carbonyl compounds and gaseous pollutants	Carbonyl, NO_2_, SO_2_ monitoring and in vitro toxicology studies	May amplify effects through airway irritation, oxidative stress, and barrier damage	Coal-source risk should not be ranked on a single toxicological endpoint	^[56,79,80]^

NSCLC: non-small cell lung cancer.

### 3.5 “中国乌蒙山肺癌”的临床与分子特征

#### 3.5.1 “中国乌蒙山肺癌”区域性临床表型

乌蒙山区燃煤污染相关肺癌的临床表型，主要表现为非吸烟女性、腺癌比例较高、发病年龄较早和多发病灶等特征。这些特征并不构成独立病理类型，而可能是长期燃煤空气暴露、共同生活环境、宿主易感性以及低剂量计算机断层扫描（low-dose computed tomography, LDCT）筛查增加后早期病灶检出增多共同作用的结果^[15,17,18,21,36,81-86]^。

宣威-富源非吸烟女性随访和滇东产煤地区住院队列研究结果^[21,36,41,85]^提示，临床分期、治疗可及性和社会经济条件也会影响区域肺癌结局。这些医院资料可补充临床表型，但不能直接用于推断人群发病风险。

2018年以来，本地系列研究多纳入宣威、富源、盘州（原盘县）等具有燃煤暴露背景的病例。相关研究结果^[17,82,83,85-93]^显示，家族肺癌患者发病较早，女性和腺癌比例较高；部分研究还观察到较高比例的早期病例，可能与高危意识增强、LDCT筛查和早期诊疗有关。

多发病灶是值得关注的区域表型。家族肺癌和宣威非小细胞肺癌（non-small cell lung cancer, NSCLC）研究^[83,94]^均观察到较高比例的多发肺结节或双侧肺多发病灶。长期燃煤污染暴露可能使多个肺区反复受损；在局部清除能力下降和宿主易感性共同作用下，不同肺区可能同步或先后出现早期分子改变。因此，多发病灶不一定代表单一病灶转移。

#### 3.5.2 “中国乌蒙山肺癌”的共病现象与全身健康线索

乌蒙地区家族肺癌研究^[82,83]^提示，高血压及部分代谢性或囊性伴随病变在家族肺癌患者中可能更常见；2026年乌蒙山区家族肺癌研究进一步显示多发肺结节和高血压比例明显升高。但这些发现主要来自医院病例资料，容易受到年龄结构、筛查强度和诊疗可及性影响。结合家庭空气污染系统综述、宣威肺癌死亡研究和美国心脏协会科学声明，家庭燃煤污染可能同时影响肺部和心血管代谢健康^[3,95-97]^。现阶段，共病现象更适合作为肺部-心血管共同损伤的探索性线索。

#### 3.5.3 “中国乌蒙山肺癌”的驱动基因谱与融合基因特征

宣威及部分具有乌蒙山区燃煤暴露背景的病例中，驱动基因谱呈现一定区域特征。研究^[84,87,94,98-100]^显示，表皮生长因子受体（epidermal growth factor receptor, *EGFR*）总突变率并不低，但经典*EGFR* 19外显子缺失和L858R比例相对较低，G719X、S768I等少见突变以及Kirsten大鼠肉瘤病毒癌基因同源物（Kirsten rat sarcoma viral oncogene homolog, *KRAS*）G12C比例较高，值得关注。

云贵高原NSCLC驱动基因研究和空气污染相关肺癌筛查研究进一步支持采用多基因检测，而非局限于单一*EGFR*位点突变检测^[84,101,102]^。

融合基因研究提示，有家庭燃煤暴露的NSCLC患者中，间变性淋巴瘤激酶（anaplastic lymphoma kinase,* ALK*）、c-ROS肉瘤致癌因子1-受体酪氨酸激酶（c-ROS proto-oncogene 1, receptor tyrosine kinase, *ROS1*）等融合事件存在特定分布。两项云南农村队列研究^[87,88]^显示，融合事件与年轻、女性、不吸烟、家族史和煤暴露等因素密切相关。这些观察仍需未来进行更大样本和统一的下一代测序（next-generation sequencing, NGS）平台验证。

对来自云贵高原相关高发地区的肺癌患者，单纯检测*EGFR* 19del/L858R可能不足以覆盖其分子异质性。标准化多基因NGS面板有助于识别少见驱动变异；对于多发结节和疑似同步多原发病例，分病灶测序还可辅助判断克隆关系。

#### 3.5.4 “中国乌蒙山肺癌”的肿瘤多组学特征

在健康暴露人群中，颊黏膜细胞基因表达分析^[59,103-105]^显示，烟煤使用者可出现炎症、凋亡和损伤修复相关改变，且与PAHs暴露的相关性显著强于PM_2.5_暴露。白细胞Alu反转座元件拷贝数研究^[52]^也提示，PAHs，尤其是5-MC，可能与基因组不稳定相关。这些可及组织证据反映了污染物进入机体后的早期分子效应。

表观遗传研究^[44,53]^进一步显示，PAHs，尤其是5-甲基香环烷，与GrimAge表观遗传年龄加速及DNA甲基化改变显著相关。肿瘤组织研究则将证据链推进至肿瘤分子特征：宣威病例可能具有较高突变负荷、特定突变特征、*EGFR*少见突变富集，以及代谢、免疫和细胞周期相关分子亚型等多组学特征^[51,106-109]^。其中，部分突变过程与烟草烟雾相关突变特征相似，但并不意味着患者具有主动吸烟暴露。

总体而言，现有证据提示“外部暴露-机体内可测量的分子效应-肿瘤分子特征”之间存在可相互衔接的证据线索。煤源和炉具决定外部污染谱，PAHs和甲基化PAHs可造成DNA损伤、基因组不稳定和表观遗传改变；长期作用后，部分人群可能形成具有区域相关驱动基因和突变特征的多组学肺腺癌（图3）。

**图3:**
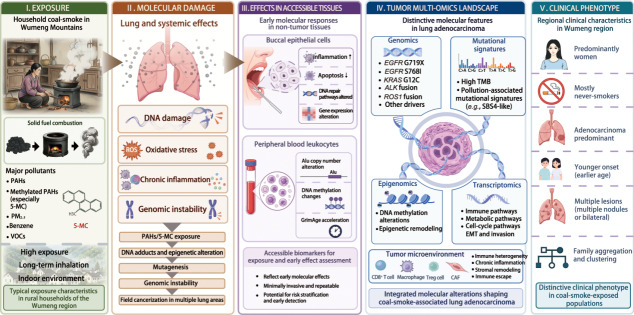
中国乌蒙山区燃煤污染相关肺癌的暴露、分子损伤、多组学与临床表型。家庭燃煤暴露可经DNA损伤、氧化应激、炎症反应和基因组不稳定等过程，影响可及组织分子效应和肿瘤多组学特征；区域性临床表型包括非吸烟女性、腺癌比例较高、较早发病、多发病灶和家族聚集倾向。

### 3.6 “中国乌蒙山肺癌”的家族聚集与遗传易感性特征

#### 3.6.1 家族聚集与胚系易感特征

乌蒙山区肺癌家族聚集不应被简单理解为单基因遗传性肺癌，也不应完全归因于共同生活环境。早期宣威家族聚集研究^[81]^发现，肺癌先证者一级亲属肺癌风险升高，女性亲属风险尤为明显，母亲风险尤其突出。值得注意的是，早期迁出宣威的家族成员肺癌发病风险仍然较高，提示遗传易感性可能参与家族聚集形成。更合理的解释是，家庭成员之间共享煤烟暴露、居住环境和生活方式，同时又可能携带不同程度的遗传易感背景。云贵高原家族肺癌研究^[82,88-90,110]^也提示，家族肺癌患者更年轻、腺癌比例更高，且转移倾向和多发病灶更突出。

胚系突变研究为共同暴露与遗传易感并存的解释提供了线索。Kanwal等^[89]^对宣威典型家族肺癌样本进行全基因组测序，并在扩展样本中验证候选基因，提出*ARHGEF5*、*MYO18B*等基因为潜在肺癌遗传易感基因。这些研究结果提示，高发家庭除共享煤烟暴露外，还可能存在影响DNA损伤修复、上皮屏障、免疫反应或污染物应答的相同遗传背景。由于样本量有限，候选基因尚不能直接用于疾病定义。

#### 3.6.2 端粒维护基因与多基因风险特征

除家系胚系突变外，宣威病例对照研究还提示端粒维护相关遗传变异值得关注。Hosgood等^[111]^联合评估痰液细胞端粒长度以及*POT1*、*TERT*和*TERF2*等端粒维护基因变异，研究结果发现：端粒长度和相关基因多态性可能影响肺癌易感性；但由于样本量较小，特定位点关联仍需在更大人群中重复验证。

跨亚洲不吸烟女性研究^[112]^进一步显示，肺腺癌多基因风险评分（polygenic risk score, PRS）和家庭用煤均与肺腺癌风险相关，但PRS的效应在用煤者中相对减弱，呈次乘性（sub-multiplicative）交互；宣威样本的附加分析也观察到相似趋势。这一结果提示，燃煤暴露与遗传易感共同参与肺癌发生，但其联合作用并非简单线性叠加。

#### 3.6.3 呼吸道微生态、免疫微环境及污染物代谢

多组学研究进一步将宿主易感性研究从“家族史”拓展至呼吸道微生态和正常肺组织基线状态。Hosgood等^[113]^发现，痰液微生物组多样性、烟煤使用和不同煤源均与呼吸道微生物组差异有关。2022年，多组学研究^[90]^进一步发现，家族背景、室内燃煤污染和肺部微生物群可能共同影响免疫和污染物代谢相关通路，并进而影响肺癌风险。

Zeng等^[83]^在43例肺癌患者正常邻近肺组织中进行RNA测序（RNA sequencing, RNA-seq），观察到家族组与散发组间存在表达的显著谱差异。由于样本量有限，且差异基因筛选采用未校正*P*<0.05，该结果更适合作为探索性线索。功能富集主要涉及先天免疫、炎症、细胞黏附和囊泡运输等过程，提示相似燃煤暴露下的组织损伤反应可能受到宿主基线状态调节。在这些正常肺组织转录组线索中，与肺上皮屏障和纤毛运动相关的MUC16信号值得进一步单独讨论。

#### 3.6.4 MUC16表达水平及纤毛功能与污染物清除相关性特征

MUC16的胚系变异、临床表达和细胞行为证据需要与纤毛功能假说区分。Wang等^[114]^在一个遗传性肿瘤家系中发现，4例肺腺癌成员共同携带3个低频*MUC16*胚系变异，且均存在多发同步肺结节。该结果提示MUC16胚系变异可能参与部分家族肺腺癌的遗传易感性，但证据来自单一家系和会议摘要，不宜直接外推。

Chen等^[115]^分析了277例NSCLC患者，并结合细胞实验发现，MUC16高表达与家族肺癌、室内空气污染和较晚分期有关，过表达可增强肿瘤细胞增殖、迁移、侵袭和顺铂耐药。该研究支持MUC16可能参与NSCLC恶性行为，但尚不能据此认定其介导燃煤污染致癌。

Zeng等^[83]^在家族肺癌正常邻近肺组织RNA-seq中发现，MUC16相关差异基因涉及纤毛组装、纤毛结构和运动等过程。结合燃煤污染暴露背景，我们提出待验证的“MUC16相关基因-纤毛结构和运动异常-肺癌高风险”假说：肺上皮纤毛功能差异可能影响污染物清除效率，从而改变肺部污染物负荷和组织损伤反应程度。该线索仍需结合暴露监测、空间组学和纤毛功能实验进一步验证。

综上，宿主易感性证据为解释“相似暴露下为何部分家庭和个体风险更高”提供了机制线索。在共同燃煤暴露背景下，遗传易感、肺部微生态和上皮屏障或纤毛功能差异可能共同调节肺部内暴露剂量和组织损伤反应，从而影响家庭聚集、多发病灶和分子异质性肺癌高发。该假说仍需在前瞻性暴露评价和功能实验中验证，宿主易感性不宜作为“中国乌蒙山肺癌”的独立定义标准。

## 4 “中国乌蒙山肺癌”的证据缺口与未来研究方向：边界验证、风险分层与精准防控

现有证据仍存在三类不足。第一，宣威-富源之外的扩展参照区缺乏统一癌症登记和个体暴露评价，区域外推仍需验证；第二，PAHs、颗粒物、矿物颗粒、金属和气态污染物之间存在共线性，单一组分的独立贡献难以区分；第三，临床分子特征和宿主易感性证据多来自医院病例，家族肺癌亚群或有限组学样本，因果机制仍需前瞻性人群研究和功能实验验证。

未来首先需要验证区域边界。扩展参照区和待验证区至少应采集四类指标：（1）县域癌症登记和死亡登记；（2）家庭燃煤类型与炉具通风情况；（3）室内外PM_2.5_、PAHs、矿物颗粒和金属混合暴露监测；（4）高危人群LDCT筛查^[91-93]^和肿瘤NGS资料。在此基础上，应整合居住年限、儿童期至成年期燃煤暴露史、临床表型和多组学资料，评价燃煤污染相关肺癌是否具有更广泛的区域扩展属性。高血压和心血管疾病等共病现象可作为未来研究拓展方向，用于探索肺部和心血管代谢损伤是否存在共同机制。

防控上可形成“暴露控制-高危识别-早筛早诊-分子分型-长期随访”的分层路径。一级预防聚焦清洁能源替代、炉具排烟改造和厨房通风改善；二级预防聚焦高危家庭LDCT筛查^[91-93]^；临床管理则结合NGS检测、多发病灶克隆关系判断和长期随访^[116]^。

总体而言，“中国乌蒙山肺癌”宜作为研究框架而非正式疾病命名使用。宣威-富源核心区已形成较完整的燃煤污染相关肺癌证据链，可作为概念提出和机制研究的主要依据；周边相似煤区可作为扩展参照，但仍需统一暴露评价、癌症登记和多组学验证。临床分子特征和宿主易感性证据为解释区域异质性、家族聚集和肺癌多发病灶提供了机制线索。该框架的公共卫生价值在于，将清洁能源替代、炉具通风、高危家庭LDCT筛查和分子分型整合为可操作的防控路径，为中国云贵高原相关高发地区肺癌精准防控提供依据^[117]^。未来需推动中国乌蒙山区及类似燃煤暴露地区肺癌研究，由单一区域经验总结转向跨区域协同验证和精准防控体系建设。我们建议建立统一的暴露评价、癌症登记、临床随访和多组学整合平台，并构建涵盖暴露控制、早筛早诊、分子分型和长期管理的区域一体化防控路径。更重要的是，我国西部地区肺癌高发地区不应只是区域性公共卫生问题的观察对象，也应成为环境暴露相关肿瘤研究、早筛早诊和精准防控体系建设的重要关注方向。通过科研证据与公共卫生实践的协同转化，有望为中国云贵高原“乌蒙山肺癌”及类似地区肺癌精准预防和综合干预提供更坚实的依据。

**专家组成员**

陈颖（云南省肿瘤医院/昆明医科大学第三附属医院/北京大学肿瘤医院云南医院），黄云超（云南省肿瘤医院/昆明医科大学第三附属医院/北京大学肿瘤医院云南医院），赵光强（云南省肿瘤医院/昆明医科大学第三附属医院/北京大学肿瘤医院云南医院），叶联华（云南省肿瘤医院/昆明医科大学第三附属医院/北京大学肿瘤医院云南医院），雷玉洁（云南省肿瘤医院/昆明医科大学第三附属医院/北京大学肿瘤医院云南医院），周永春（云南省肿瘤医院/昆明医科大学第三附属医院/北京大学肿瘤医院云南医院），姚宏（云南省肿瘤医院/昆明医科大学第三附属医院/北京大学肿瘤医院云南医院），杨政鸿（云南省肿瘤医院/昆明医科大学第三附属医院/北京大学肿瘤医院云南医院），曾敬统（云南省肿瘤医院/昆明医科大学第三附属医院/北京大学肿瘤医院云南医院），李继华（曲靖市疾病预防控制中心），宁伯福（宣威市疾病预防控制中心），梁好（四川大学华西医院肺癌中心），范亚光（天津医科大学总医院肺癌研究所），黄开利（四川大学华西医院肺癌中心），邓汉宇（四川大学华西医院肺癌中心），胡佳（四川大学华西医院肺癌中心），龚会（中国西部肺癌研究协作中心），舒晓玲（中国西部肺癌研究协作中心），范雪娇（四川大学华西医院肺癌中心），朱大兴（四川大学华西医院肺癌中心），罗猛（贵州省人民医院），田林玮（香港大学公共卫生学院），周清华（四川大学华西医院肺癌中心）
